# Raman Spectroscopy Applications in Grapevine: Metabolic Analysis of Plants Infected by Two Different Viruses

**DOI:** 10.3389/fpls.2022.917226

**Published:** 2022-06-14

**Authors:** Luisa Mandrile, Chiara D’Errico, Floriana Nuzzo, Giulia Barzan, Slavica Matić, Andrea M. Giovannozzi, Andrea M. Rossi, Giorgio Gambino, Emanuela Noris

**Affiliations:** ^1^Istituto Nazionale di Ricerca Metrologica (INRIM), Torino, Italy; ^2^Institute for Sustainable Plant Protection, National Research Council of Italy (CNR), Torino, Italy

**Keywords:** Raman scattering, *Vitis vinifera*, carotenoids, virus, early diagnosis

## Abstract

Grapevine is one of the most cultivated fruit plant among economically relevant species in the world. It is vegetatively propagated and can be attacked by more than 80 viruses with possible detrimental effects on crop yield and wine quality. Preventive measures relying on extensive and robust diagnosis are fundamental to guarantee the use of virus-free grapevine plants and to manage its diseases. New phenotyping techniques for non-invasive identification of biochemical changes occurring during virus infection can be used for rapid diagnostic purposes. Here, we have investigated the potential of Raman spectroscopy (RS) to identify the presence of two different viruses, grapevine fan leaf virus (GFLV) and grapevine rupestris stem pitting-associated virus (GRSPaV) in *Vitis vinifera* cv. Chardonnay. We showed that RS can discriminate healthy plants from those infected by each of the two viruses, even in the absence of visible symptoms, with accuracy up to 100% and 80% for GFLV and GRSPaV, respectively. Chemometric analyses of the Raman spectra followed by chemical measurements showed that RS could probe a decrease in the carotenoid content in infected leaves, more profoundly altered by GFLV infection. Transcriptional analysis of genes involved in the carotenoid pathway confirmed that this biosynthetic process is altered during infection. These results indicate that RS is a cutting-edge alternative for a real-time dynamic monitoring of pathogens in grapevine plants and can be useful for studying the metabolic changes ensuing from plant stresses.

## Introduction

Grapevine (*Vitis vinifera* L.) is one of the most important fruit crop, with up to 7 million hectares cultivated worldwide in 2020 (FAOSTAT, 2020). Grapevine is mainly grown for wine production and for fresh and dry fruit consumption, but it is also used for seed oil extraction, alcoholic beverage and vinegar production; moreover, several social, touristic, and cultural activities are linked to its cultivation, generating a positive impact on the economy.

Grapevine is affected by several pathogens, including fungi, oomycota, phytoplasmas, and viruses heavily influencing yield and quality of the crop and reducing the economic revenues. Among grapevine pathogens, viruses are widespread in all cultivated areas, causing different diseases, such as the rugose wood complex, leafroll, infectious degeneration, and fleck disease (Fuchs, 2020). Up to now, more than 80 viruses from 17 families and 34 genera have been identified (Martelli, 2014, 2018), frequently occurring in mixed infection.

Within this large number of viral entities threatening grapevine, grapevine rupestris stem pitting-associated virus (GRSPaV) and grapevine fanleaf virus (GFLV) are two well-known and widespread examples. After its discovery about two decades ago, GRSPaV is nowadays considered one of the most ubiquitous viruses, found in Europe, America, Australia, and Asia (Meng and Rowhani, 2017). GRSPaV belongs to the genus *Foveavirus*, family *Betaflexiviridae*, and it is generally associated to “Rupestris Stem Pitting,” a disorder of the “Rugose Wood complex” (Meng and Gonsalves, 2003). Its presence has been linked to other grapevine diseases, including the vein-clearing complex on cv. Chardonnay (Lunden et al., 2009). Nonetheless, in most cases GRSPaV induces latent infections, with no visible symptoms on infected plants. Despite this, GRSPaV was reported to trigger a number of transcriptional changes on cv. Bosco, mainly regarding photosynthesis and CO_2_ fixation, leading to a moderate decrease of the photosynthetic process and an altered reaction of plants to biotic/abiotic stress, underlying possible beneficial effects mediated by this virus toward abiotic factors (Gambino et al., 2012; Pantaleo et al., 2016; Tobar et al., 2020).

GFLV (family *Secoviridae*, genus *Nepovirus*) is a harmful and economically deleterious virus, responsible for the “Grapevine infectious degeneration” complex (Sanfaçon et al., 2009). Symptoms induced by GFLV include vein yellowing, mosaics, internode shortening, typical leaf deformations, smaller and fewer bunches, with irregular ripening. The variability of symptoms observed in vineyards depends on the virus strain, grapevine genotype, cultural practices, and environmental conditions (Martelli, 2017). GFLV is transmitted by the soil-borne ectoparasitic nematode *Xiphinema index* and by infected plant material. Beside phenotypic alterations typical of infectious degeneration, the physiological and molecular changes induced by GFLV can be occasionally associated to an improved tolerance toward fungal infections (Gilardi et al., 2020) and to a moderate water stress (Krebelj et al., 2022). Overall, GRSPaV and GFLV represent two virus models regarding the symptomatology induced on vine plants, which interact with the host in complex and unexpected ways, justifying to more deeply explore the changes occurring during the infection processes.

Early diagnosis of plant pathogens is crucial for a proper disease management, allowing not only to eliminate infected material and reduce further spread of the pathogens, but also to implement clean stock programs useful to preserve the sanitary status of a crop. This is particularly relevant for grapevine, a vegetatively propagated perennial crop, and for viral pathogens which cannot be eliminated with chemical pesticides. For these, in fact, eradication programs are required before the nursery stage and during the clonal selection, currently performed applying sanitation techniques such as meristem culture, thermotherapy, and somatic embryogenesis. Specifically, due to the extensive use of clonal multiplication of grapevine, many countries have established strict regulations for the grapevine propagation material, in order to verify the presence of viruses and reduce the risk of disease spread (Golino et al., 2017). Plant disease diagnosis is commonly performed using molecular-based procedures (Fang and Ramasamy, 2015; Martinelli et al., 2015), which can be time-consuming, unsuitable for rapidly testing large numbers of samples, require skilled personnel and the availability of pathogen-specific reagents (gene sequences or antibodies), and are not frequently implemented for field application. Indeed, grapevine certification schemes mainly rely on serological and molecular assays, aided by biological indexing, time-consuming and expensive activities often requiring multiple evaluations. In Italy, sanitary schemes dictate that all materials test negative for grapevine virus A (GVA), GFLV, Arabis mosaic virus (ArMV), grapevine leafroll-associated virus-1 and -3 (GLRaV-1, −3), and grapevine fleck virus (GFkV, this only for rootstocks; Italian regulation D.M. 7 July 2006 and D.L. 02 February 2021). Therefore, new diagnostic tools, ideally suitable for field testing of plants by untrained personnel, using friendly and inexpensive equipment and providing results in a short time, with minimal number of steps would be extremely important. Such strategies could allow extensive and fast screening of imported vegetative material, preventing disease spread.

Raman spectroscopy (RS) records the molecular vibrations of cellular metabolites present in a specimen in the absence of labels or reagents and has been recently proposed as a non-destructive and rapid diagnostic procedure for plant pathogens. The spectra obtained from healthy and diseased plant samples are used as specific fingerprints, reflecting changes in cellular metabolites occurring following infection by pathogens or during abiotic stresses. Indeed, several groups including our laboratory have shown that RS can sense the presence of different plant pathogens, among which viruses, in different cultivated crops (Yeturu et al., 2016; Egging et al., 2018; Farber and Kurouski, 2018; Farber et al., 2019a,b; Sanchez et al., 2020). In particular, we showed that specific changes in tomato plants artificially inoculated with two different viruses can be identified by RS, at a stage when visual symptoms were not yet visible (Mandrile et al., 2019).

In the current study, we investigated the potential of RS to determine the occurrence of two different viruses infecting grapevine cv. Chardonnay; the two pathogens were chosen as examples of a latent-asymptomatic virus (GRSPaV) and a dangerous-symptomatic virus (GFLV), whose absence is required in the certification protocols. Plants separately infected by the two viruses were analyzed with a Raman microscope apparatus at different time points during the vegetative season and systemic molecular changes induced by the viruses were analyzed by quantitative reverse transcription-PCR (RT-qPCR).

## Materials and Methods

### Plants

*V. vinifera* cv. Chardonnay plants infected by either GFLV (cluster IB; NCBI Acc. No. MN889891) or GRSPaV (phylogenetic group GRSPaV-SG1; NCBI Acc. No. MN889892) were previously described in Gilardi et al. (2020). In this work, 2-year-old infected plantlets and healthy individuals (*n* = 4) were maintained in 5-L pots filled with a peat substrate (TS4, Turco Silvestro, Italy). Plants were kept under a gauze greenhouse for the whole duration of the experiment, with constant watering. Each plant represents a biological replica.

### RNA Extraction and RT-qPCR

Total RNA was extracted using a rapid CTAB method (Gambino et al., 2008) and its quantity and quality were evaluated with a NanoDrop 1000 spectrophotometer (Thermo Fisher Scientific, Waltham, MA, United States). RNA was then treated with DNase (DNase I, Thermo Fisher Scientific, Waltham, MA, United States) and reverse-transcribed using the High-Capacity cDNA Reverse Transcription Kit (Thermo Fisher Scientific), following manufacturer’s instructions.

RT-qPCR reactions were performed in a CFX Connect Real-Time PCR system (Bio-Rad Laboratories, Hercules, CA, United States), using SYBR Green (SensiFAST™ SYBR® No-ROX Kit; Meridian Bioscience, Memphis, Tennessee, United States) with the following cycling conditions: denaturation at 95°C for 2 min, followed by 40 cycles at 95°C for 15 s and 60°C for 30 s. RT-qPCR was conducted for the relative quantification of GFLV and GRSPaV, using primers specific for viral RdRp (Gilardi et al., 2020), and for transcriptional analysis of genes representative of the carotenoid pathway, using Ubiquitin (*VvUBI*) and Actin1 (*VvACT1*) as internal controls. The primers for RT-qPCR are listed in Supplementary Table S1. Four independent biological replicates and three technical replicates were run for each RT-qPCR. Gene expression data were subjected to analysis of variance (ANOVA), followed by the Tukey’s HSD *post hoc* test (*p* ≤ 0.05). The SPSS statistical software package (SPSS Inc., Cary, NC, United States, v.23) was used to run statistical analyses.

### Raman Spectroscopic Measurements

Raman spectra were acquired from one half of the fifth leaf counting from the apex, while the other half was used for virus detection and transcript accumulation analysis. Leaf samples for RS analysis were stored in plastic bags and kept on ice until spectra acquisition within the following 4 h. Spectra (400–3,100 cm^−1^; 5 cm^−1^ resolution) were acquired using a Dispersive Raman Spectrometer (DRX Thermo Fisher Scientific, Waltham, United States; 785 nm excitation laser, 10× microscope objective, 2 μm laser spot diameter, 10 mW laser power; 20 scansions, 1 s each), were collected on the same point of the leaf, taking three points per leaf, on three different leaf lobes.

The spectrometer was weekly calibrated using a certified white light for intensity and neon gas lines for frequency. Moreover, a Si standard was measured before each session, to guarantee consistency within measurements and to avoid differences due to instrument performances. Four different measurements were performed, at monthly intervals, starting in May, until August 2021 (T1 to T4).

### Chemometric Analysis of Raman Spectra

Chemometric analysis was conducted using the PLS Toolbox (Eigenvector Research, Inc., Manson, WA) for Matlab R2015a (Mathworks, Natick, MA). Spectral range between 650 and 3,060 cm^−1^ was considered. Spectra pre-processing consisted in smoothing (Savitzky-Glay filter, 21 pt.), baseline correction (automatic weighted least square regression, second order and Whittaker filter with asymmetry 1e^−5^, λ 1,000), and mean centering. Principal Component Analysis (PCA) was used to find non-random data structures attesting non-random variability between groups of spectra. The effect of the different factors of the experimental design was evaluated by analysis of variance simultaneous component analysis (ANOVA-SCA, also known as ASCA), considering the following k factors: (i) “time” (T1, T2, T3, and T4); (ii) “virus” (presence of infection; levels healthy, GRSPaV and GFLV), and (iii) “biological replicates” (levels: different plant specimens).

ASCA was performed considering the two-way correlations between factors. The significance of the experimental factors was quantified determining values of *p* through a permutation test between the levels of the factors (Zwanenburg et al., 2011). The H0 hypothesis of no experimental effect, indicating no difference between the levels averages of the effect matrices, with a confidence level of p was tested. Values of *p* were obtained for the main effects by randomizing the levels of each factor under consideration.

Partial least squares discriminant analysis (PLS-DA) was finally used as a classification method to test the possibility to recognize infected plants. Since an external test set for validation was not available, leave-one plant-out cross validation (CV) was used to determine the classification error (CE).

### Sample Extraction and Analysis of Total Carotenoids, Chlorophylls, and Polyphenols

Plant extracts were prepared according to Alrifai et al. (2021), with slight modifications. Grapevine leaves were freeze-dried and maintained at −80°C; 25–30 mg of powdered material were extracted in 4 ml of an acetone:ethanol (1:1, v/v) solution and extracts were sonicated in a water bath for 15 min and incubated at room temperature for 4 h, with shaking at 400 rpm (Sky4 Shaking Incubator, Argo Lab). After centrifugation (1,600 ×*g*, 5 min), each supernatant was transferred to a clean tube; pellets were re-extracted twice with the same solvent, once using 2 ml for 2 h, followed by 1 ml for 1 h. Supernatants from the same sample were pooled. For total carotenoid and chlorophyl content analysis, a 250-μl aliquot of each extract was added in triplicate to a 96-well microplate. The plate was analyzed immediately using a UV/VIS Varioskan Lux (Thermo Fisher Scientific, Waltham, United States) multi-wells reader, measuring absorbance at 452 nm. A calibration curve was prepared with a β-carotene (Sigma Aldrich, Certified Reference Material, >99%) solution, using at least five concentrations from 2 to 50 μg/ml, *R*^2^ > 0.99. Total carotenoid content was expressed as μg of β-carotene equivalents/g of dry weight sample. Absorbance at 666 nm was also recorded to evaluate the chlorophyll content and relative comparison between the tested samples was performed to provide semi-quantitative information.

Total polyphenol content was measured by the Folin–Ciocalteu method, using the same ethanol:acetone (1:1) leaf extracts (see above). Aliquots of 200 μl of each extract were added to 15-ml tubes containing 3 ml ultrapure water and 200 μl Folin–Ciocalteu reagent (Sigma Aldrich). After mixing and incubating the samples for 6 min at room temperature, 200 μl of 20% (w/v) Na_2_CO_3_ (Carlo Erba) were added to each tube and vortexed. After 30 min incubation at 37°C, aliquots of 200 μl of each sample were placed in triplicate in a 96-wells microplate and absorbance at 765 nm was measured with the UV/VIS Varioskan Lux multi-wells reader, by subtracting the absorbance of the blank (ethanol:acetone solution, 1:1). A calibration curve made with gallic acid was used as standard, measuring at least five concentrations from 40 to 200 mg/L. Results were normalized to the weight of the dried leaf sample (mg/L).

## Experimental Results and Discussion

### Virus Accumulation in Grapevine Plants Along the Vegetative Season

In this work, we considered grapevine plants cv. Chardonnay infected by either GRSPaV or GFLV, and healthy control individuals (Gilardi et al., 2020). Plants were surveyed along the whole vegetative season from May to August 2021, at four different time points (T1 to T4) at monthly intervals. During the whole season, no visible symptoms could be detected on plants infected by either virus, in agreement with a previous report (Gilardi et al., 2020) and in line with unpublished observations of young plants kept in pots, across several years (G. Gambino, personal observations).

**Figure 1:**
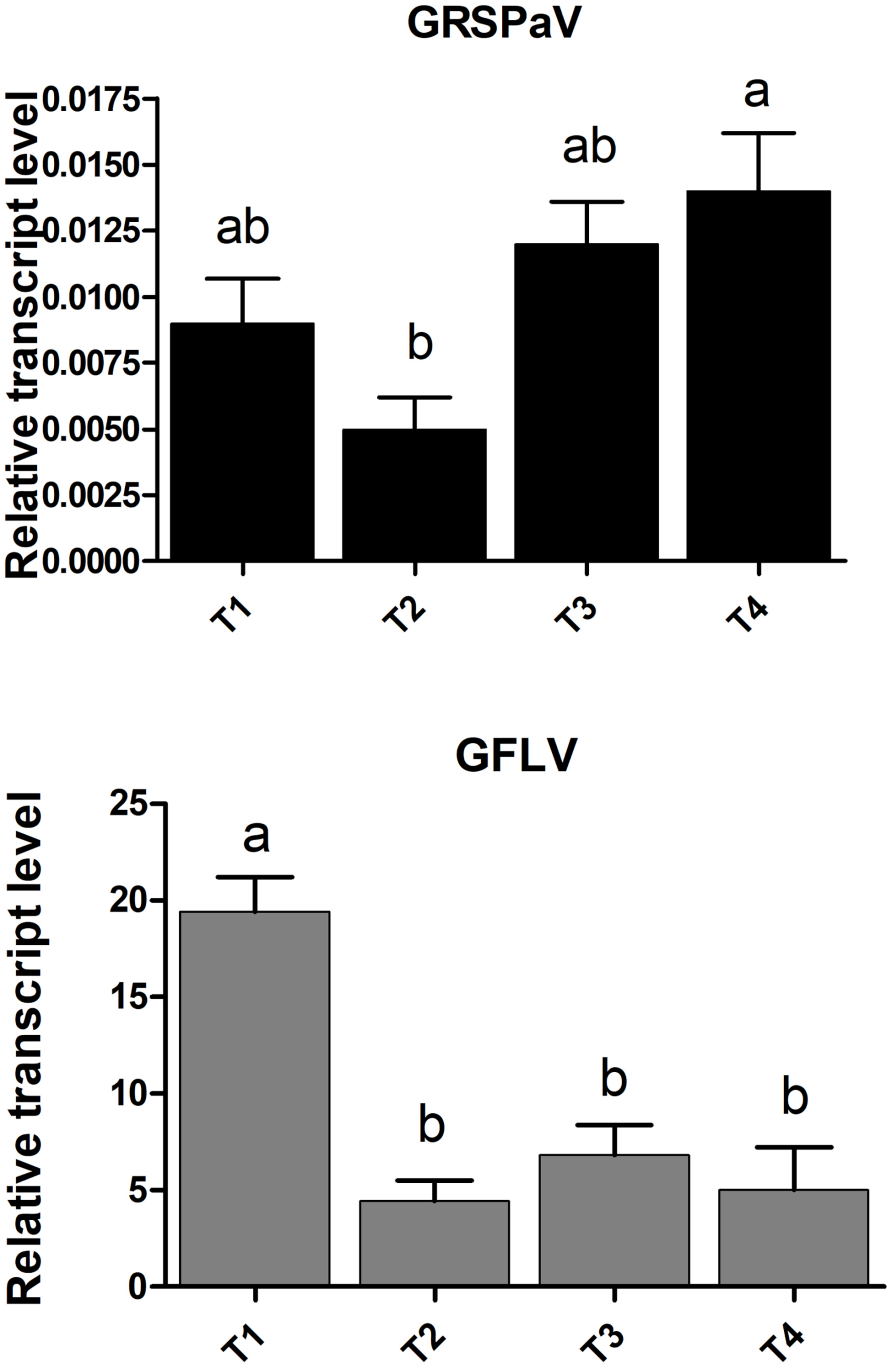
Relative accumulation of grapevine rupestris stem pitting-associated virus (GRSPaV) and grapevine fan leaf virus (GFLV) in grapevine cv. Chardonnay leaf tissue, at different times (T1 to T4, at monthly intervals) during the vegetative season. Quantitative reverse transcription-PCR (RT-qPCR) signals were normalized to *VvAct* and *VvUBI* transcripts. Data are presented as the mean ± SE (*n* = 4). Lowercase letters denote significant differences attested by Tukey’s honestly significant difference (HSD) test (*p* < 0.05).

RT-qPCR virus quantification analysis showed an overall stable accumulation of GRSPaV along the whole duration of the experiment, with a slight increase only at the end of the season (Figure 1). In vineyard conditions, the GRSPaV titer in leaves tends to decrease as the season progresses, while no such decrease occurred in the present conditions (Gambino et al., 2012). On the contrary, a remarkable drop in the accumulation of GFLV occurred since the second time point analyzed (T2, June), with no further changes during the vegetative season (Figure 1). The reduction of the GFLV titer along the season is in line with observations recorded in vineyard, where the highest GFLV concentrations in leaves were found in May, i.e., at the beginning of the vegetative period (Krebelj et al., 2015; Gilardi et al., 2020).

### Raman Spectra Measurements of Leaves

**Figure 2:**
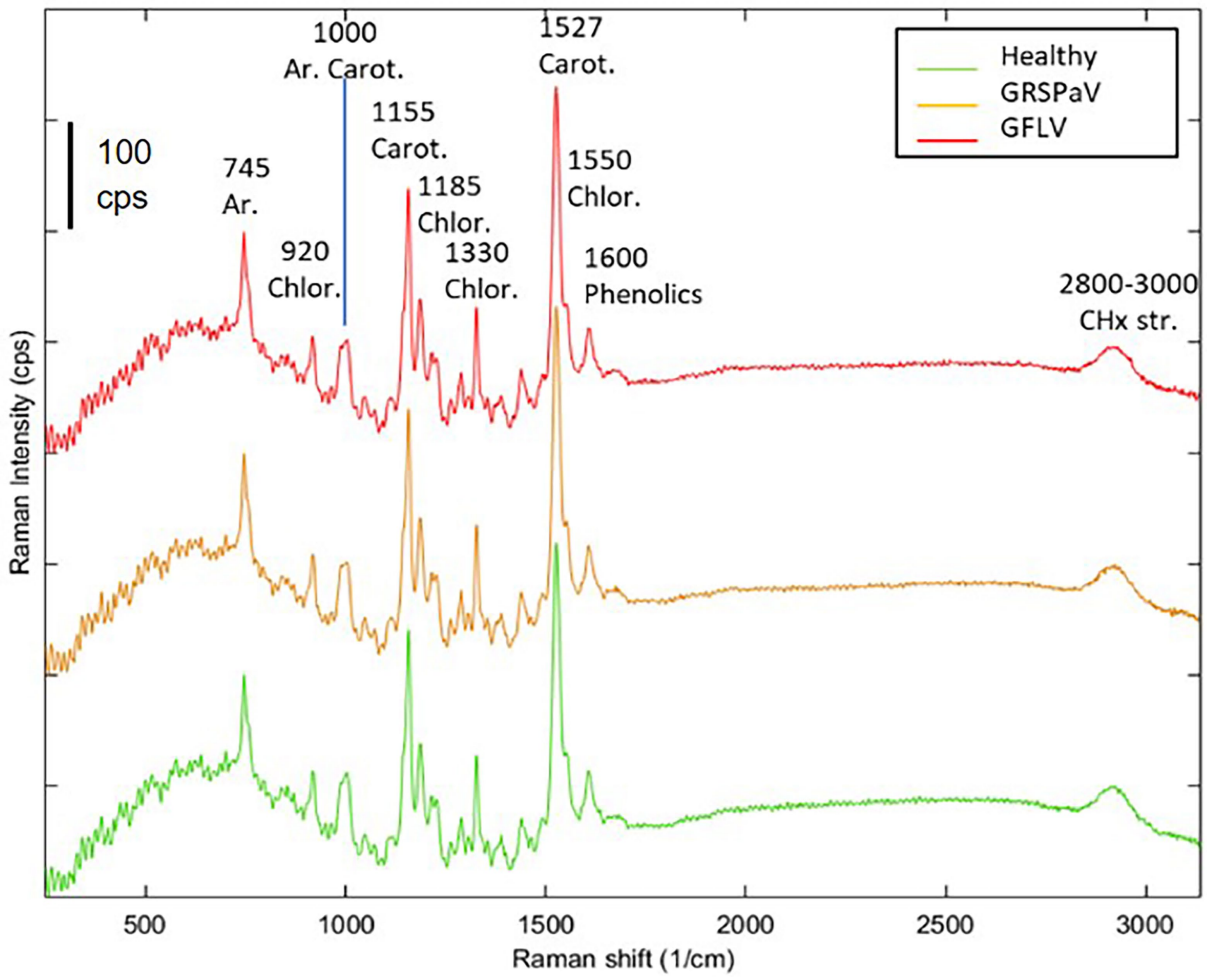
Average Raman spectra of healthy (green), GRSPaV- (yellow), and GFLV- (red)-infected grapevine cv. Chardonnay leaves. Spectra are the result of four plants per group. Representative spectra collected in the first measurement session (T1) are shown.

The Raman spectra of grapevine leaves were collected on intact plant material, focusing the excitation laser directly onto the leaf surface. A near infrared laser wavelength was used to limit the undesired fluorescence effect disturbing Raman signals. Other research paper dealing with Raman measurements on plant tissues report the alternative use of 785 nm (Dou et al., 2021), 830 nm (Farber et al., 2019b; Sanchez et al., 2020; Payne et al., 2022), or 1,064 nm (Yeturu et al., 2016; Farber and Kurouski, 2018; Skoczowski et al., 2022) laser wavelengths to minimize fluorescence interference and increase signal-to-noise ratio. At the same time, a relatively low laser power and low magnification objective were adopted to avoid thermal stress of the tissue and to collect information from a relatively large area (spot size >2 μm). The mean spectra of grapevine leaves showed vibrational bands that were assigned to cellulose, carotenoids, polyphenols, chlorophylls, xylan, lignin, and proteins, being the major components of leaves (Figure 2). The assignment of bands of the most relevant peaks are reported in Table 1. According to previous literature, most of the wavenumbers were related to photosynthetic pigments (Zeng et al., 2021).

**Table 1:** Raman bands assignments for grapevine leaves.

Band (cm^−1^)	Vibrational assignment	References
2,800–3,000	CH_x_ stretching	
1,605	m ν(phenyl ring; phenolics and lignin)	Eravuchira et al., 2012
1,551	m br chlorophyll - central 16-membered-ring vib. + ν(C=C; pyrrole ring)	
1,526	s ν1(C–C; carotenoids)	Koyama et al., 1986
1,483	m δ(CH_2_) and δ (CH_3_)	
1,438	m ν(phenyl ring; phenolics)	Eravuchira et al., 2012
1,370	δCH_2_ bending vibration (aliphatic)	Yu et al., 2007
1,328	m δ(CH) + ν(CN; pyrrole ring br.—chlorophylls)	Boldt et al., 1987
1,320	[δ(C12 − H), ν(C11-C12)](β-carotene)	Eravuchira et al., 2012
1,280	m δ(phenyl-OH; phenolics) + − δ(CH). ν(CN; chlorophyll)	Eravuchira et al., 2012; Yeturu et al., 2016
1,215	m δ(CH) + δ(CH_2_; chlorophyll)	Boldt et al., 1987
1,180	ms ν(CC) + γ(CH; chlorophylls) + δ(CH phenyl; phenolics)	Boldt et al., 1987; Eravuchira et al., 2012
1,150	s ν2(C\\C; carotenoids)	Gill et al., 1970
1,140	m sh ν(CN). δ(CNC; chlorophyll)	Boldt et al., 1987
1,110	δ (C – OH; carbohydrates)	Farber and Kurouski, 2018
1,050	ν (C – O) + ν(C – C) + ν(C – OH; carbohydrates)	Farber and Kurouski, 2018
1,000	m δ(C – CH_3_; carotenoids)	Gill et al., 1970
980	m undefined (chlorophylls)	
909	m undefined (chlorophylls)	
738	ms ring br. mode (aromatics)	

Following this analysis, the spectra obtained from healthy plants were compared with those collected from virus-infected plants, at the different time points. Similar spectral profiles were registered among the three different groups of samples, at the different time measurements (Figure 2), indicating that, at preliminary observation, the spectral fingerprint of leaves was not severely influenced by the presence of virus infection, but only minimal changes were registered. The entire fingerprint regions of the mean spectra for the three classes of plants and the four sampling times are shown in supporting information (Supplementary Figure S1) for a better comparison. For the majority of bands, frequency mismatches between healthy and infected plants can be noticed since the third sampling time.

**Figure 3:**
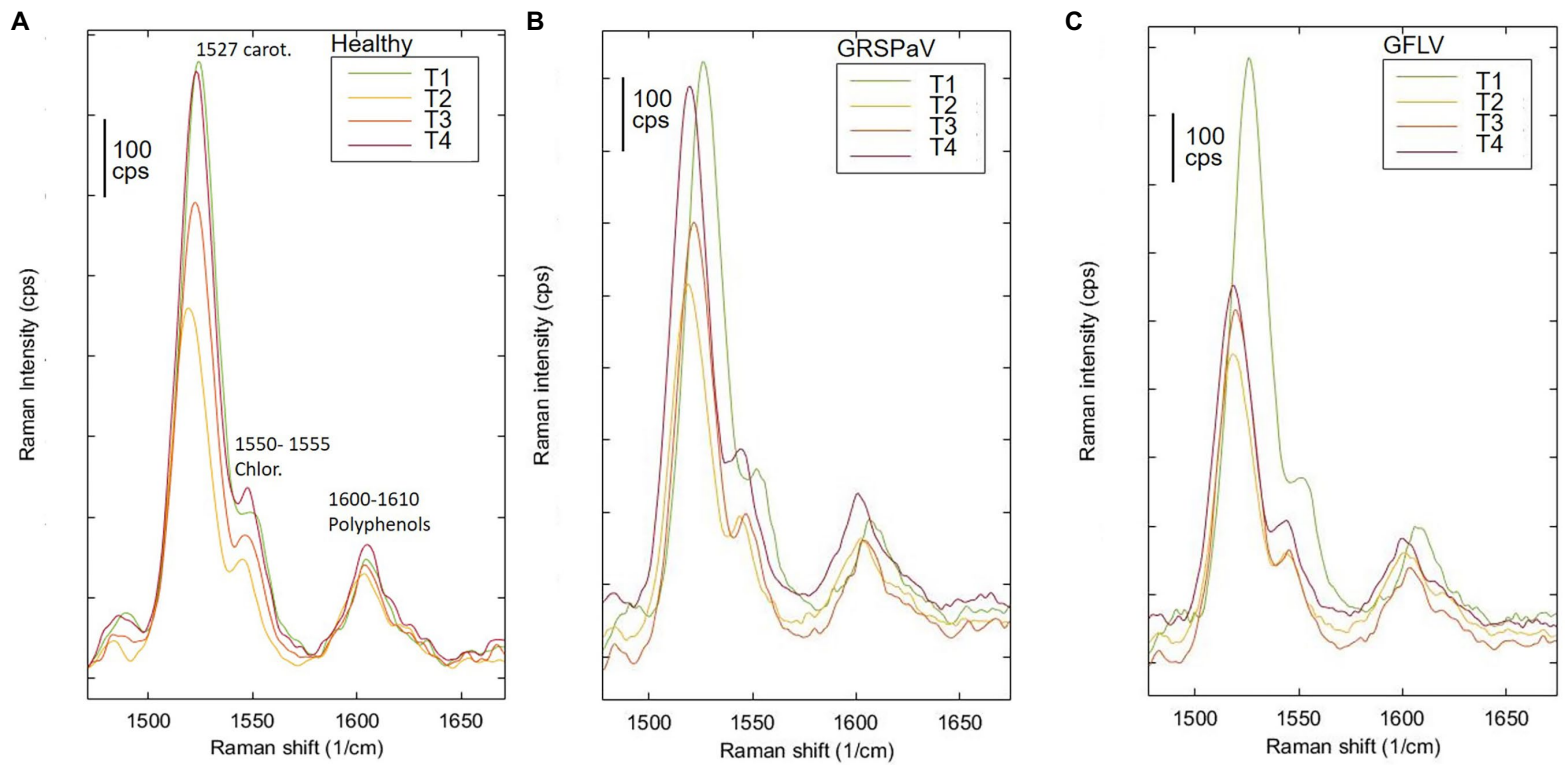
Raman spectroscopic analysis of **(A)** healthy and of **(B)** GRSPaV- or **(C)** GFLV-infected grapevine cv. Chardonnay leaves, at different time points (T1 to T4) during the vegetative season. Focus on the peaks associated to carotenoids and chlorophyll. Average spectra are the result of four plants per group.

Previous works have determined the assignment of Raman bands obtained from leaf samples which are mostly due to carotenoids, being among the most Raman active classes of compounds present in such tissue (Yeturu et al., 2016). In particular, the most evident peak observed at 1,526 cm^−1^ is assigned to the stretching of the –C=C– double bond in the conjugated chain of carotenoids (Adar, 2017), while the shoulder at 1,550 cm^−1^ is due to chlorophylls. Focusing our attention on this particular band and comparing the mean spectra of healthy and infected plants monitored during the entire vegetative season, a reduced carotenoid concentration in leaves of GFLV-infected plants was noticed since the second measurement (T2). On the contrary, no such tendency occurred in healthy plants or in GRSPaV-infected plants (Figure 3). In addition, a frequency change that exceeds the resolution limit of 5 cm^−1^, was registered in infected tissues for the carotenoid peak, as well as for other bands in the Raman fingerprint region, since the second sampling (Figure 3). In particular, the –C=C– stretching shifted to a slightly lower frequency in infected plants (from 1,526 to 1,518 cm^−1^ for GFLV and from 1,526 to 1,520 cm^−1^ for GRSPaV, at T3), possibly resulting from a modification of the carotenoids profile occurring in these plants (Figure 3). A previous study by Withnall et al. (2003) showed a linear inverse dependency of the frequency location of the band of –C=C– double bonds and the length of the conjugated chain of carotenoids. However, due to the intrinsic limits of Raman measurements on complex biological matrices, the available data do not allow to specifically address the accumulation of carotenoid molecules of a specific length, an issue which should be investigated with more selective techniques.

Overall, the modification of the Raman peaks, especially those associated to carotenoids, provides an indication that the infection by these two viruses leads to a different metabolic response of infected plants. In particular, a reduced concentration of carotenoids in grapevine suggests a functional link to either a modulation of transcripts involved in carotenoid metabolism or to their degradation and fragmentation or conversion to apocarotenoids, i.e., signaling molecules produced in response to stress. A decrease in carotenoid concentration has been frequently reported when analyzing by Raman spectroscopy plants infected by pathogens (Dou et al., 2021; Farber et al., 2021; Vallejo-Pérez et al., 2021) or subjected to abiotic stresses (Altangerel et al., 2017; Sng et al., 2020), confirming the role of this class of molecules in plant stress responses.

Beside the visual comparison of the average spectra collected from healthy and infected plants over time, a more complete investigation regarding the changes in the Raman profiles was conducted, with a multivariate unsupervised visualization method. This procedure allows to consider the whole spectral information and to test the significance of spectral differences within the groups included in the experimental design. For this, the entire dataset was processed with ASCA using the four plants present in each group (factor “Infection,” levels “healthy,” “GRSPaV,” “GFLV”), considering one leaf per plant, three spectra per leaf, four sampling sessions over four measurements, at monthly intervals (factor “Time,” levels “T1,” “T2,” “T3,” and “T4”). This process is expected to model the effect of each of the factors included in the experimental design and to evaluate the significance of each effect. At the same time, a PCA model was calculated for each design factor, to help visualizing the results. Then, the significance of each factor was tested by permutation tests within the levels of the factors, providing a *p* < 0.5 value for significant factors. Unfortunately, the ASCA model for the combined dataset showed that no significant spectral variation could be modeled over time to distinguish the three levels of the factor “infection” (Table 2). On the contrary, the factors “time” and “plant specimen” resulted significantly different.

**Table 2:** Results of ANOVA simultaneous component analysis (ASCA) elaboration on the complete data set of samples.

Factor	No. of principals components	Effect	*p*
Time	3	39.76	0.001
Plant specimen	11	13.57	0.001
Virus	2	2.77	1.00
Mean	-	0.00	-
Residuals	-	52.28	-

*Results were obtained from 144 spectra collected from 12 plants, over 4 months.*

**Figure 4:**
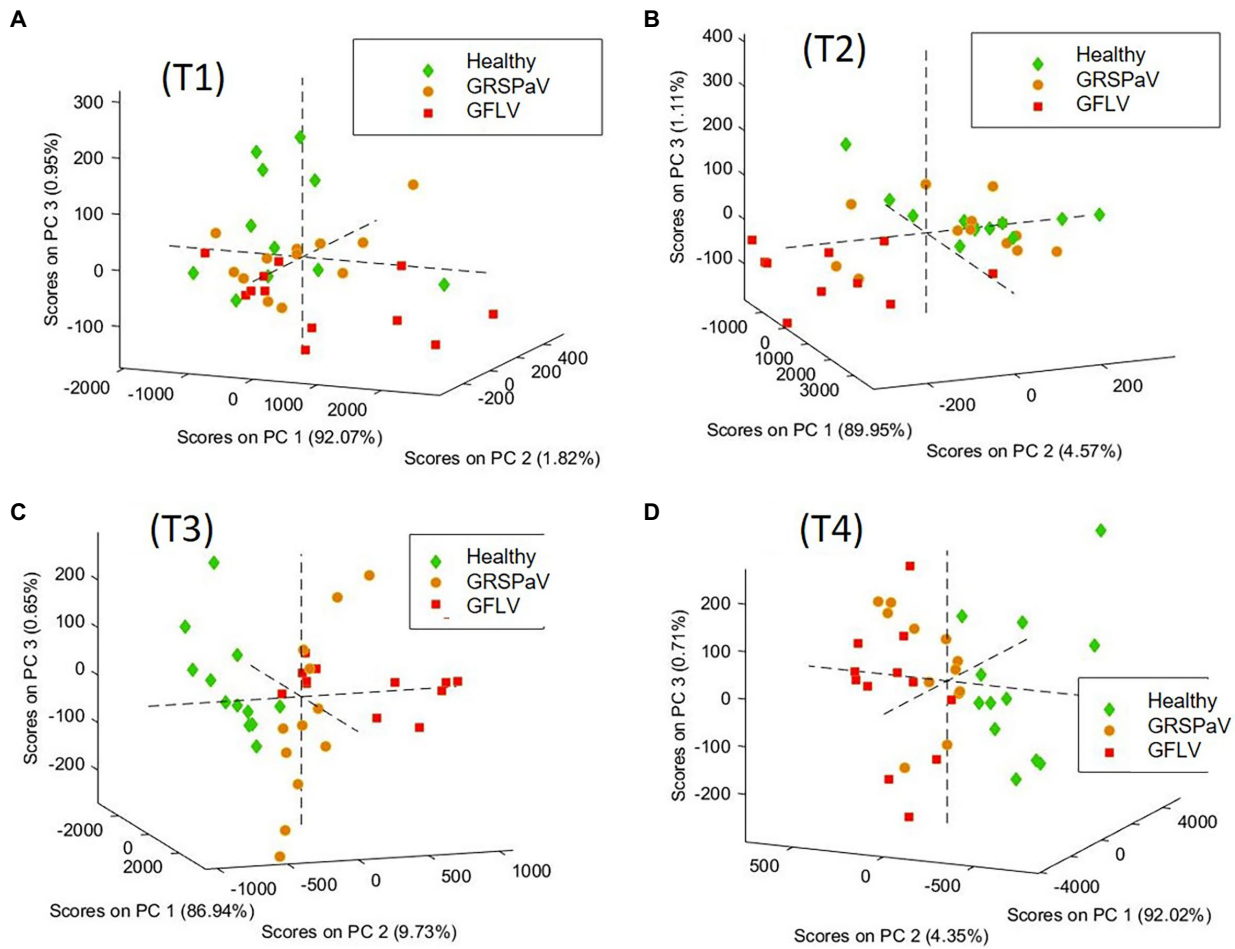
PCA score plots of the spectra of healthy and GRSPaV- or GFLV-infected grapevine cv. Chardonnay plants, calculated at (**(A)** T1, **(B)** T2, **(C)** T3, **(D)** T4, at monthly intervals). 3D graph rotation is set to optimize result visualization.

These results urged us to consider separately the four sampling sessions and to determine the discrimination ability of RS to detect molecular changes induced in leaves by virus infection, on a temporal basis. For this, in order to obtain data grouping in accordance with the infection, at each sampling time, a PCA was performed, i.e., a common visualization method used to reduce the number of variables and to plot multivariate data as a scatter plot accounting for unsupervised agglomeration of samples due to common features. The PCA score plots obtained are shown in Figure 4, colored according to the infection condition at each sampling time.

In order to elucidate the spectral features driving this unsupervised clustering of spectra, the loadings of the different PCA models were compared. In details, at T3 and T4, the loadings of the first three PCs are very similar (Supplementary Figure S2). Noteworthy, the most important features allowing to separate the different spectra are PC1, which refers to the overall spectral intensity, mainly regarding carotenoid peaks, and PC2, accounting to the band shifts observed at 1,527 cm^−1^ (carotenoids) and 780 cm^−1^ (aromatics, probably mainly phenolics, such as anthocyanin). This analysis confirms that the differences found in the mean spectra are common to all spectra of the same group, albeit with different magnitude. Moreover, this procedure showed that at T3 and T4 it is possible to distinguish healthy plants from those infected by the two different viruses. On the contrary, at T1 and T2, no score grouping could be obtained in the PC1, 2, 3 scores plot, indicating a poor differentiation of spectral profiles of healthy and infected plants. The variance captured at T1 and T2 by the first three PCs, which is mainly related to the fingerprint region between 500 and 1,600 cm^−1^ does not drive clear grouping of scores related to the infection conditions of the samples (Supplementary Figures S2A,B).

### Supervised Data Analysis

Considering the absence of visible symptoms induced on grapevine by the two viruses here considered, a major goal of this work was to determine if RS coupled to multivariate statistical methods could discriminate healthy plants from infected individuals. Therefore, PLS-DA was used as a classification method to evaluate the possibility to discriminate healthy from infected plants based on their Raman spectra. Due to the reduced number of plants included in the experimental design which could not be separated into a calibration and a validation set, the Leave-one-group-out cross-validation (CV) method was used; noteworthy, to test the validity of the model with a method more similar to external set testing, full leave-one-out CV was avoided, and the exclusion groups of CV corresponding to “one-plant-out” at a time were set. Therefore, to test the recognition ability of RS, different class vectors were considered, as follows: (1) three class models (healthy, GRSPaV, GFLV) to simultaneously distinguish healthy plants from plants infected by each of the viruses, (2) two class models (healthy vs. infected plants), considering all infected plants together, and (3) two class models (healthy vs. GRSPaV-infected plants or healthy vs. GFLV-infected plants), separately considering the two different viruses. The classification results of such a cross-validation test are reported in Table 3.

**Table 3:** PLS-DA classification to distinguish grapevine plants infected by either GFLV or GRSPaV from healthy individuals, over the vegetative season.

Model		T1 (%)	T2 (%)	T3 (%)	T4 (%)
3 Classes	(H,R,G)	50	52	14	19
2 Classes	(H,I), I = R + G	19	36	8	8
2 Classes	(H,R)	25	31	8	12
2 Classes	(H,G)	13	17	0	0

Results are expressed as classification error (CE) and cross validation (CV). H, healthy; R, GRSPaV; G, GFLV; and CE, classification error.

Although in the first two measurements (T1 and T2) it was not possible to discriminate the presence of either GRSPaV or GFLV in the plants with a high level of accuracy in CV, infected plants could be distinguished with a classification error (CE) < 20% starting from the T3 measurement. In particular, infected plants (considering GRSPaV and GFLV together) could be distinguished from healthy individuals with CE values of 8% at T3 and T4, a result particularly relevant considering the complete absence of symptoms. Noteworthy, CE 0% were obtained for GFLV-infected tissue in the last two sampling times, probably resulting from changes in the metabolism of carotenoids occurring in such plants, justifying further investigations, as below described.

**Figure 5:**
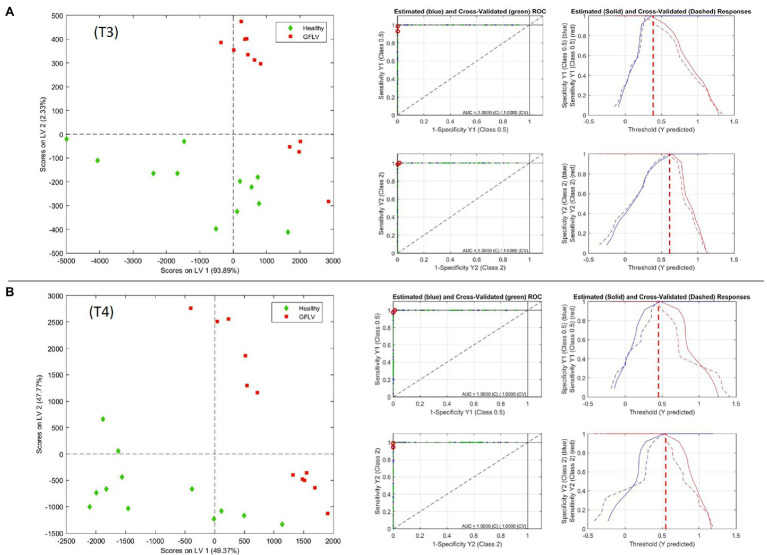
PLS-DA model at T3 **(A)** and T4 **(B)**, with scores on latent variable 1 and 2 plots and Receiver Operating Characteristic (ROC) measurements for GFLV recognition from healthy plants.

The score plots of the two best models in the area defined by the two first latent variables (LVs) of the PLS-DA model and the Receiver Operating Characteristic (ROC) curves are shown in Figure 5, providing a clear visual indication of the model sensitivity and specificity. The two relevant LVs of these models are shown in Supplementary Figure S3, while the model images for the three classes (H,R,G) and for (H,R) at T3 and T4 are reported in Supplementary Figure S4.

Interestingly, the discriminative ability of RS was independent from the amount of virus determined in the leaves and was higher toward the end of the vegetative season (Table 3; Figure 1). This is particularly interesting in the case of GFLV for which the best classification rates in the PLS-DA model were calculated at the T3-T4 measurements against the backdrop of a sharp viral load reduction in the same period. Nonetheless, this result can be assessed in the light of a “load metabolic effect” induced by virus infection in this crop along the seasonal progression (Gambino et al., 2012; Chitarra et al., 2018; Martin et al., 2021). Moreover, the results here reported support previous observations of a higher metabolic impact on grapevine plants exerted by GFLV compared to GRSPaV, corroborating the concept of a co-evolution of GRSPaV with this crop (Gambino et al., 2012) possibly resulting from the long-lasting presence of a hard to eradicate pathogen in grapevine.

### Validations *via* Chemical Analytical Extractions

**Figure 6:**
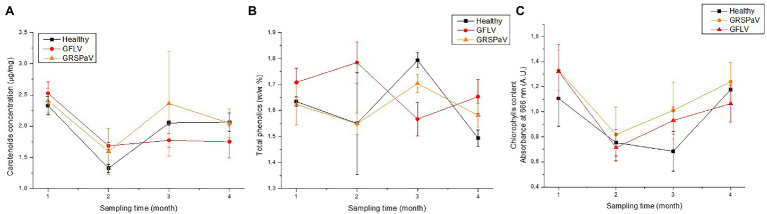
Accumulation of **(A)** carotenoid, **(B)** phenolic, and **(C)** chlorophyll compounds in healthy and infected grapevine leaf samples, during the vegetative season. The values reported are mean ± SE of the classes of compounds obtained from four independent biological samples (*n* = 4).

To confirm the results of the RS analyses, the concentration of the three main classes of pigments, i.e., carotenoids, total phenolics, and chlorophylls, were measured by spectrophotometric assays in the same tissues used for RS. As it can be observed in Figure 6, the peculiar trends measured with Raman spectroscopy concerning the concentration of carotenoids were confirmed. In particular, a decrease in carotenoid concentration can be noticed from T1 to T4 in GFLV-infected plants (Figure 6A), in accordance with the RS results (Figure 3). Regarding the other two classes of compounds investigated, i.e., chlorophylls and polyphenols, no significant trends are revealed, in line with the observation that their Raman signals were not relevant for the discrimination between healthy and infected plants. However, interestingly, significant differences in the content of total phenolics compounds between healthy and GFLV-infected plants were recorded at T3 and T4, probably supporting the higher discrimination accuracy for infected plants.

Regarding chlorophylls, a similar trend was detected over time in all groups of plants, independently on the presence of virus infection. Based on these results, the accumulation of chlorophylls does not seem to be influenced by the infection process, rather by the environmental conditions, while the content of carotenoid and phenolic compounds is altered in infected plants. This observation is in line with recent studies highlighting the relevance of secondary metabolites as players in plant defense responses, thus underlying the importance of characterizing the metabolic profiles associated to disease susceptibility traits in grapevine as a promising approach to identify trait-related biomarkers (Maia et al., 2020).

### Transcriptional Analysis of Genes Involved in the Carotenoid Pathway

**Figure 7:**
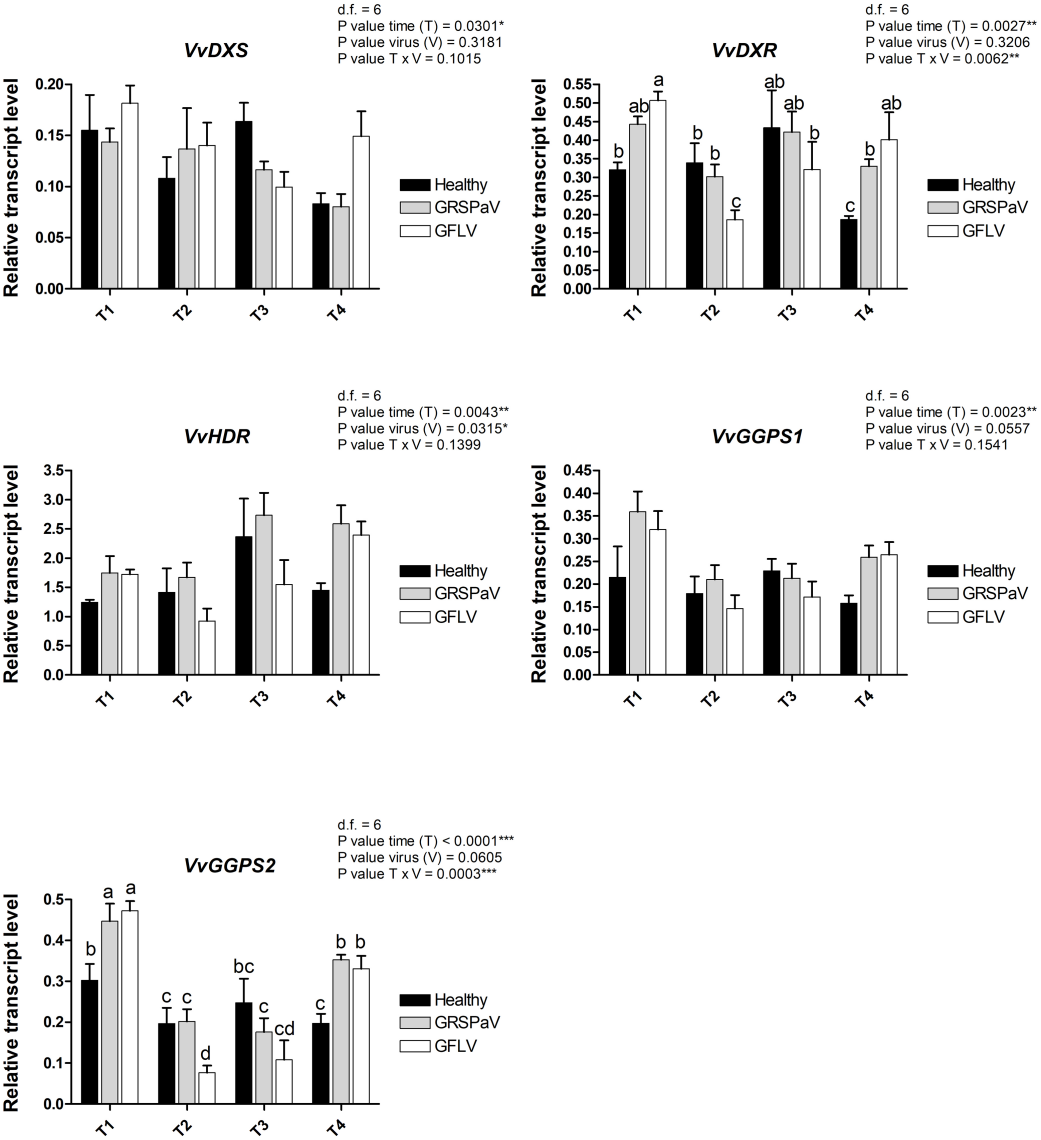
Relative expression levels of *VvDXS* (VIT_05s0020g02130), *VvDXR* (VIT_17s0000g08390), *VvHDR* (VIT_03s0063g02030), *VvGGPS1* (VIT_04s0023g01210), and *VvGGPS2* (VIT_18s0001g12000), measured by RT-qPCR. Samples were collected in four sampling points along the season (May_T1, June_T2, July_T3, and August_T4). RT-qPCR signals were normalized to *VvAct* and *VvUBI* transcripts. Data are presented as the mean ± SE (*n* = 4). Significance of sampling time, virus, and time x virus (T × V) interaction was assessed by Tukey’s HSD test for *p* ≤ 0.05 (*), *p* ≤ 0.01 (**), *p* ≤ 0.001 (***) and the corresponding results are given above each graph in the figure panel. Lower case letters above bars are reported when the T × V interaction are statistically significant as attested by Tukey’s HSD.

Since the most interesting information related to virus infection determined by RS is linked to the carotenoid content, a transcriptional study was conducted by RT-qPCR to measure the expression level of a set of target genes involved in carotenoid metabolism (Leng et al., 2017). Carotenoids are mainly synthesized from isopentenyl diphosphate (IPP) and dimethylallyl diphosphate (DMAPP) produced through the monoterpene biosynthetic pathway (MEP). In particular, we tested the first two genes of the biosynthetic MEP route, 1-deoxy-D-xylulose-5-phosphate synthase (*VvDXS*) and 1-deoxy-D-xylulose-5-phosphate reductoisomerase (*VvDXR*), and one of the last genes, 1-hydroxy-2-methyl-2-(E)-butenyl-4-diphosphate reductase (*VvHDR*). For these genes, a slight transcript modulation occurred in both healthy and virus-infected plants. While the sampling time (T) was significant for all the three genes, the effect of virus (V) was significant only in the case of *VvDXR*, whose expression increased at T4 in GRSPaV- and GFLV-infected plants. The interaction between virus and time (V × T) was significant only for *VvDXR*, showing a decrease in GFLV-infected plants at T2, followed by an increase at T4 in both virus-infected samples (Figure 7).

Two isoforms of geranyl pyrophosphate synthase (*VvGPPS*), a gene operating along the MEP pathway, responsible for the production of geranyl pyrophosphate acting as substrate of monoterpenes synthases in the late carotenoid pathway, resulted strongly transcriptionally regulated along with time progression (T), but not by the presence of virus infection (V). In addition, considering the V × T interaction, a significant downregulation of *VvGPPS2* was recorded in particular in GFLV-infected plants at T2 (Figure 7), mirroring the carotenoid reduction observed by Raman analysis (Figure 3).

**Figure 8:**
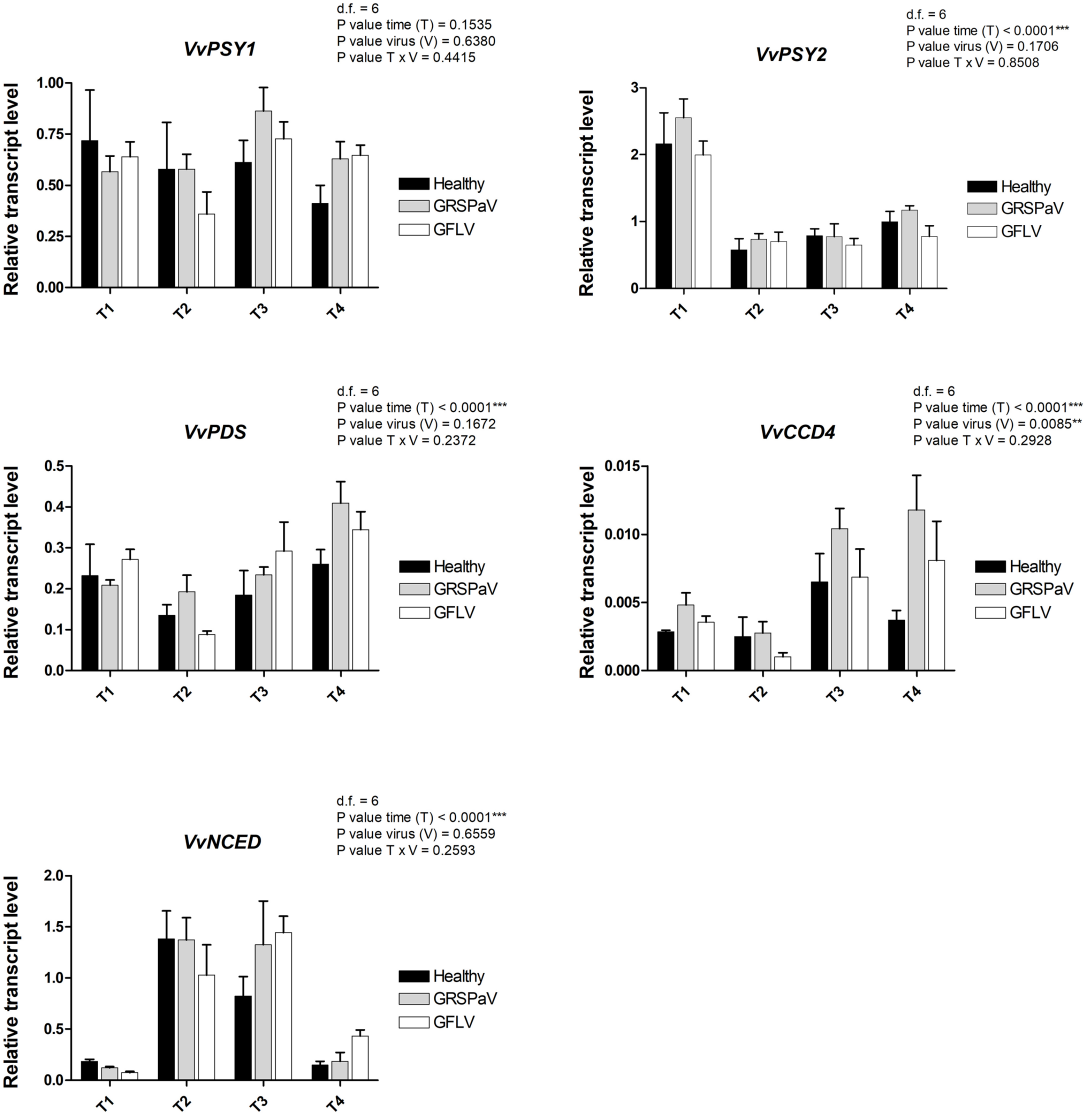
Relative expression levels of *VvPSY1* (VIT_04s0079g00680), *VvPSY2* (VIT_12s0028g00960), *VvPDS* (VIT_09s0002g00100), *VvCCD4* (VIT_02s0087g00930), and *VvNCED* (VIT_19s0093g00550), measured by RT-qPCR. Samples were collected in four sampling points along the season (May_T1, June_T2, July_T3, and August_T4). RT-qPCR signals were normalized to *VvAct* and *VvUBI* transcripts. Data are presented as the mean ± SE (*n* = 4). Significance of sampling time, virus, and time x virus (T × V) interaction was assessed by Tukey’s HSD test for *p* ≤ 0.01 (**), *p* ≤ 0.001 (***) and the corresponding results are given above each graph in the figure panel.

Of the two genes encoding the phytoene synthase (*VvPSY*), considered as a bottleneck reaction in the carotenoid pathway, *VvPSY1* did not show any significant modulation regarding the effects of virus infection or time progression, while *VvPSY2* showed a strong T effect (Figure 8), indicating its prominent role in the carotenoid reduction occurring after the T1 sampling in the whole set of samples (Figure 3). The phytoene produced by *VvPSY* is then desaturated through the action of phytoene desaturase (*VvPDS*) which showed a modulation affected only by T, in particular at T4.

Among the genes involved in carotenoid catabolism, we analyzed a carotenoid cleavage dioxygenase (*VvCCD4*) and a 9-cis-epoxycarotenoid dioxygenase (*VvNCED*). *VvCCD4* is linked to the production of volatile compounds and strigolactones and showed significant V and T effects, with a negative correlation with the accumulation of carotenoids at T3 and T4. On the other side, *VvNCED*, a key enzyme in the biosynthesis of abscisic acid (ABA), showed a significant T effect with negative correlations with the carotenoids at T2 and T3, and an interesting upregulation in GFLV-infected plants at T4 (Figure 8).

Collectively, positive correlations between the reduced accumulation of carotenoids, particularly, in GFLV-infected plants, and the downregulation of transcripts involved in their biosynthesis (i.e., *VvGGPS2* and *VvPSY2*) were detected, accompanied by an upregulation of genes responsible for carotenoid catabolism, i.e., *VvCCD4* and *VvNCED*. This suggests that virus infection, particularly in the case of GFLV, can accelerate the natural reduction of photosynthetic processes mediated by carotenoids occurring across the vegetative season. Moreover, it indicates that RS can sense a metabolic stress response leading to the accumulation of ABA and strigolactones (Milborrow and Lee, 1998; Auldridge et al., 2006; Havaux, 2013), originating from carotenoid precursors.

## Conclusion

A growing number of evidences are showing that RS techniques represent a non-invasive, non-destructive analytical approach to monitor the sanitary status of plants (Payne and Kourowsky, 2021). Here, we applied RS to grapevine, one of the most economically important crops worldwide, affected by relatively higher number of pathogens compared to other fruit trees and subjected to strict certification programs to guarantee its phytosanitary status. The PLS-DA model here obtained from the RS data demonstrated the suitability of the RS approach to discriminate healthy from infected plants, even in the absence of macroscopic symptoms, with up to 92% accuracy for GRSPaV and 100% accuracy for GFLV, the latter taken as a representative virus that should be absent in certified virus-free plant materials. The Raman spectra allowed to identify the major metabolic changes occurring in this crop in response to virus infection, paving the way to adopt a RS-based approach as a complementary procedure to detect early stages of viral infection not only in vineyards but also in the nurseries. Following proper verification of the congruence of the results, direct evaluation of plants grown in vineyards will be feasible using high-throughput portable Raman spectrometers, as reported by other groups (Farber and Kurouski, 2018; Krimmer et al., 2019; Sanchez et al., 2019; Gupta et al., 2020).

## Data Availability Statement

The raw data supporting the conclusions of this article will be made available by the authors, without undue reservation.

## Author Contributions

LM, CDE, SM, FN, and GB performed the experiments. LM, EN, GB, GG, AMG, and FN analyzed the data. LM, GG, and EN wrote the manuscript. LM, GG, AMR, and EN conceived the study and participated in its design. All authors contributed to the article and approved the submitted version.

## Funding

The present work has been supported by the Fondazione Cassa di Risparmio di Torino, Project ViraDEP, ref. no. 2020.0598.

## Conflict of Interest

The authors declare that the research was conducted in the absence of any commercial or financial relationships that could be construed as a potential conflict of interest.

## Publisher’s Note

All claims expressed in this article are solely those of the authors and do not necessarily represent those of their affiliated organizations, or those of the publisher, the editors and the reviewers. Any product that may be evaluated in this article, or claim that may be made by its manufacturer, is not guaranteed or endorsed by the publisher.
