# Supplementary material for: Raman Spectroscopy Applications in Grapevine: Metabolic Analysis of Plants Infected by Two Different Viruses
Source: Front Plant Sci. 2022 Jun 14;13:917226. doi: 10.3389/fpls.2022.917226 (PMC9239551; doi:10.3389/fpls.2022.917226)
Supplement: Supplementary file 1 [file Data_Sheet_1.pdf]

SUPPLEMENTARY MATERIAL

Raman spectroscopy applications in grapevine: metabolic analysis of plants infected by two different viruses

Luisa Mandrile[1], Chiara D'Errico[2], Floriana Nuzzo[2], Giulia Barzan[1], Slavica Matic[2], Andrea M. Giovannozzi[1], Andrea M. Rossi[1*], Giorgio Gambino[2], Emanuela Noris[2*]

[1]Istituto Nazionale di Ricerca Metrologica (INRIM), Torino, Italy

[2]Institute for Sustainable Plant Protection, National Research Council of Italy (CNR), Torino, Italy

Supplementary Table S1. List of the oligonucleotides used in this study.

Target (Gene ID)	*Gene abbreviation*	Gene Description	Primer	Primer sequences 5'-3'	References
VIT_05s0020g02130	*VvDXS*	1-deoxy-D-xylulose-5-phosphate synthase, chloroplast precursor	Forward	CTGTACGCTCTCATTTCCTGC	Battilana et al., 2011
			Reverse	TGATACTCCTCCCTATCCGAAAG	
VIT_17s0000g08390	*VvDXR*	1-deoxy-D-xylulose 5-phosphate reductoisomerase	Forward	AGAGGCTTTGGCTGACTGTGA	Martin et al., 2012
			Reverse	AACCTGCGCAACCTACTATTCC	
VIT_03s0063g02030	*VvHDR*	1-hydroxy-2-methyl- 2-(E)-butenyl-4-diphosphate reductase	Forward	TCTTCCTCGTCTGTGGCTGTT	Martin et al., 2012
			Reverse	GCGATTCATGAGCTCCAGAGT	
VIT_04s0023g01210	*VvGGPS1*	geranylgeranyl pyrophosphate synthase 1, chloroplastic-like	Forward	TCCGTCCCGTTCTCTGTATC	Leng et al., 2017
			Reverse	CCTTGTGATTTGTGGGCTTT	
VIT_18s0001g12000	*VvGGPS2*	geranylgeranyl pyrophosphate synthase 2, chloroplastic-like	Forward	GGAACAAAGACGCCACATTT	Leng et al., 2017
			Reverse	AGCCTCGTCCAAGGCTTTAT	
VIT_04s0079g00680	*VvPSY1*	phytoene synthase 1, chloroplastic-like	Forward	GTTGGGTTAATGAGCGTTCCA	This manuscript
			Reverse	CTCCGTGGTTGCCTGTGAT	
VIT_12s0028g00960	*VvPSY2*	phytoene synthase 2, chloroplastic-like	Forward	TGGGATAGCCAATCAGCTCACT	This manuscript
			Reverse	ATTCTTCCCCTCCTAGCATCCT	
VIT_09s0002g00100	*VvPDS*	phytoene desaturase	Forward	TTGTGCACAGGCTATTGTAAAGG	This manuscript
			Reverse	CCTCGGCCAACTTTTGTTCTC	
VIT_02s0087g00930	*VvCCD4*	carotenoid cleavage dioxygenase 4	Forward	GCCCCAACCCCCAGTTC	Gambino et al., 2012
			Reverse	GCATGCCATCACCATCAAAG	
VIT_19s0093g00550	*VvNCED*	9-cis-epoxycarotenoid dioxygenase	Forward	GGTGGTGAGCCTCTGTTCCT	Ferrero et al., 2018
			Reverse	CTGTAAATTCGTGGCGTTCACT	
NC_001948		Grapevine rupesris stem pitting-associated virus	Forward	GTGATCCATGTCAAAGCACATATG	Pantaleo et al., 2016
			Reverse	CTCAGCGCCCAAAATTGC	
MN889891		Grapevine fanleaf virus	Forward	TGGCACAGTCTGTCATGCAA	Gilardi et al., 2020
			Reverse	CAAACTTGGCCATCTGCAACT	
VIT_04s0044g00580	*VvACT*	Actin	Forward	TCCGTTCTCAGAGATCAACAA	Gambino et al., 2012
			Reverse	ACTCTCTCATCTCAAGATATTCTATGG	
VIT_16s0098g01190	*VvUBI*	Ubiquitin	Forward	TCTGAGGCTTCGTGGTGGTA	Gambino et al., 2012
			Reverse	AGGCGTGCATAACATTTGCG	

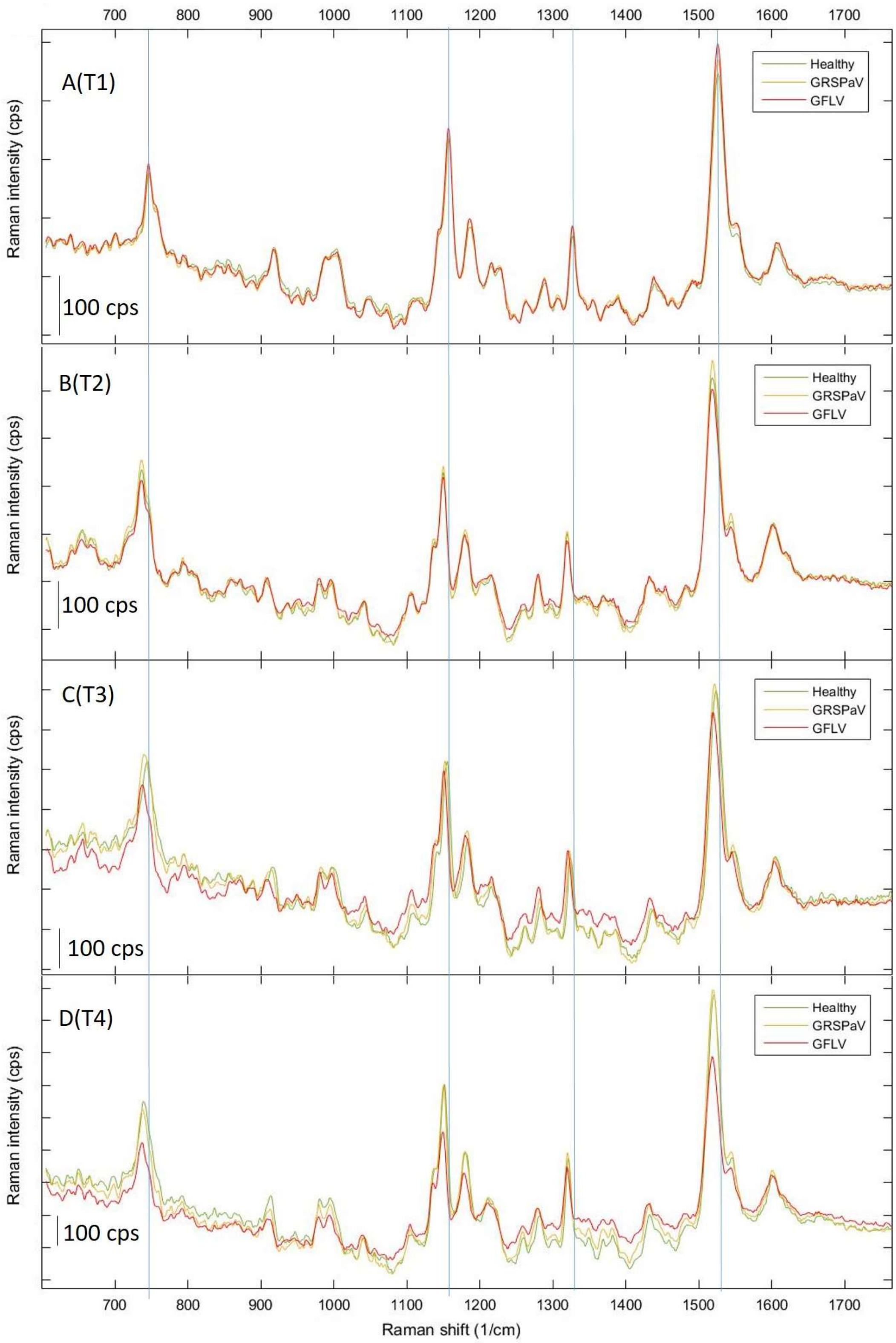

Figure S1. Mean Raman spectra from 600-1750 cm^{-1} of healthy and GRSPaV- or GFLV-infected grapevine cv. Chardonnay plants, at monthly intervals: (A) T1, (B) T2, (C) T3, and (D) T4.

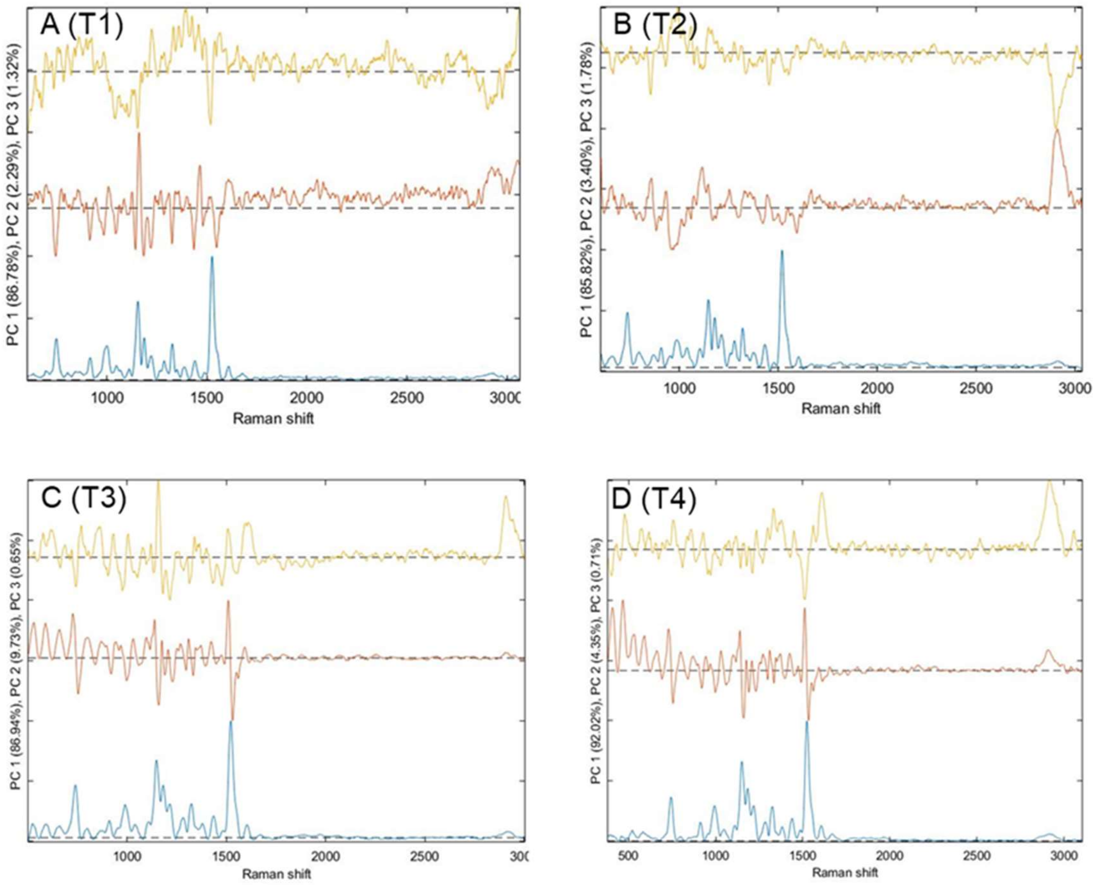

Figure S2. Loadings of the three first relevant PCs of the PCA models, calculated for each sampling time, i.e. (A) T1, (B) T2, (C) T3, and (D) T4, at monthly intervals.

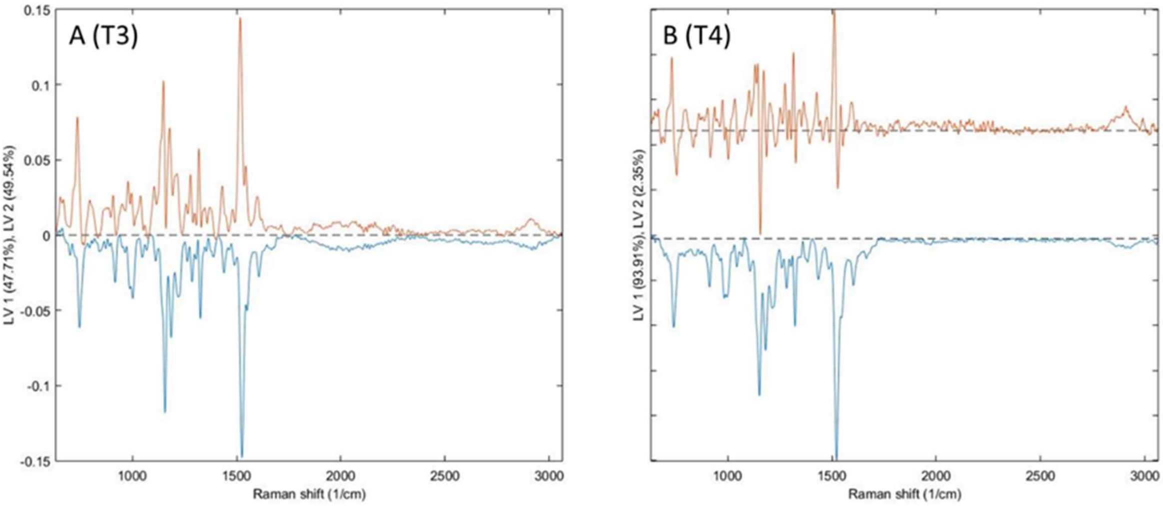

Figure S3. Loadings of the latent variables of PLS-DA for GFLV recognition, at the T3 (A) and T4 (B) measurements.

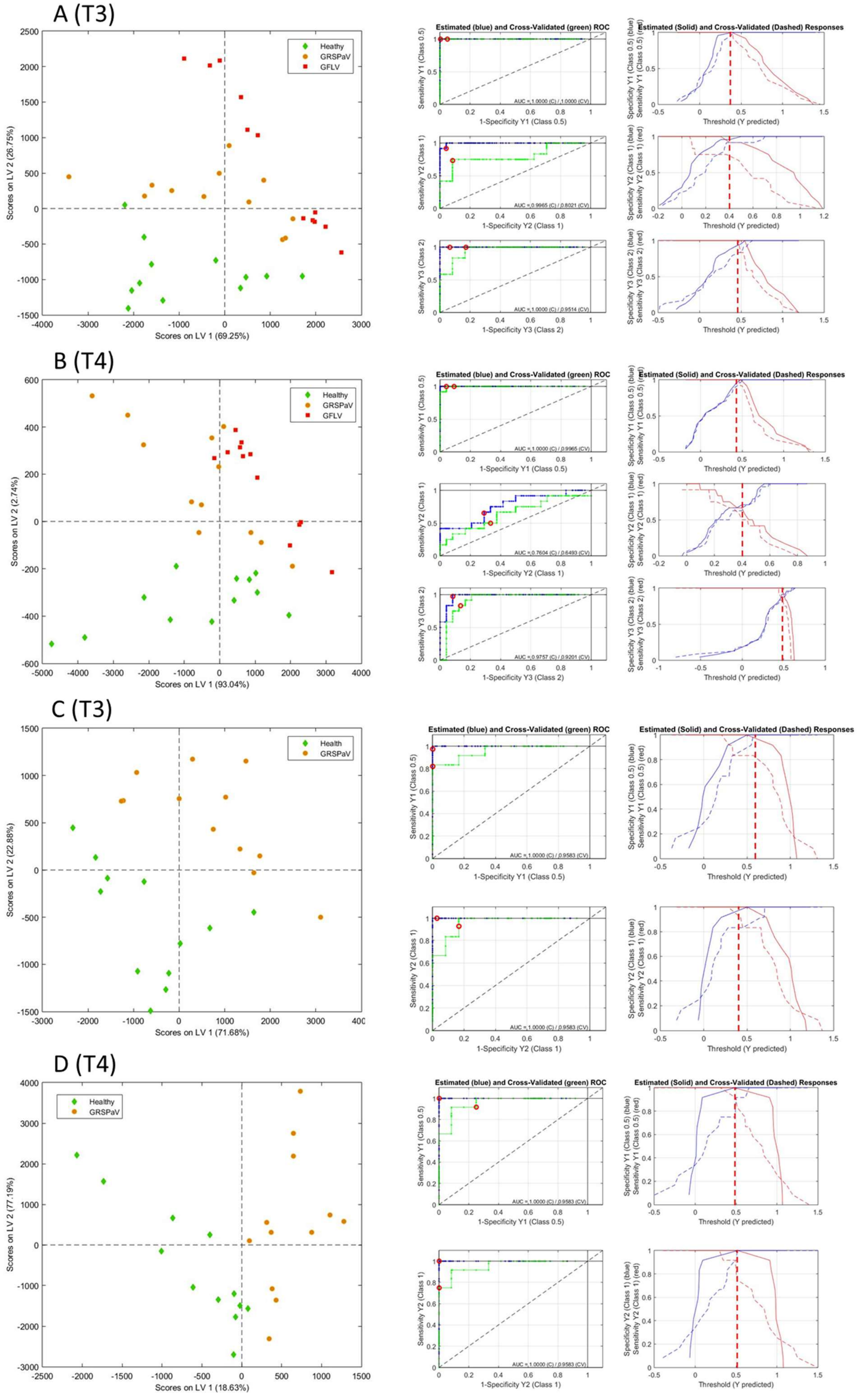
A (T3)
B (T4)
C (T3)
D (T4)
Healthy
GRSPaV
GFLV
Health
Scores on LV 1 (69.25%)
Scores on LV 2 (26.75%)
Scores on LV 1 (93.04%)
Scores on LV 2 (2.74%)
Scores on LV 1 (71.68%)
Scores on LV 2 (22.88%)
Scores on LV 1 (18.63%)
Scores on LV 2 (77.19%)
Estimated (blue) and Cross-Validated (green) ROC
Estimated (Solid) and Cross-Validated (Dashed) Responses
Sensitivity Y1 (Class 0.5)
1-Specificity Y1 (Class 0.5)
Sensitivity Y2 (Class 1)
1-Specificity Y2 (Class 1)
Sensitivity Y3 (Class 2)
1-Specificity Y3 (Class 2)
Threshold (Y predicted)
Specificity Y1 (Class 0.5) (blue)
Sensitivity Y1 (Class 0.5) (red)
Specificity Y2 (Class 1) (blue)
Sensitivity Y2 (Class 1) (red)
Specificity Y3 (Class 2) (blue)
Sensitivity Y3 (Class 2) (red)
AUC = 1.0000 (C) / 1.0000 (CV)
AUC = 0.9965 (C) / 0.8021 (CV)
AUC = 1.0000 (C) / 0.9514 (CV)
AUC = 1.0000 (C) / 0.9965 (CV)
AUC = 0.7604 (C) / 0.6493 (CV)
AUC = 0.9757 (C) / 0.9201 (CV)
AUC = 1.0000 (C) / 0.9583 (CV)

Figure S4. Scores of the latent variable 1 and 2 plots and Receiver Operating Characteristic (ROC) of the PLS-DA model for the simultaneous classification of healthy, GFLV and GRSPaV, at the T3 (A) and T4 (B) measurements and for the classification of GRSPaV and healthy plants, at the T3 (C) and T4 (D) measurements.

Supplementary References

Battilana, J., Emanuelli, F., Gambino, G., Gribaudo, I., Gasperi, F., Boss, P. K., et al. (2011). Functional effect of grapevine 1-deoxy-D-xylulose 5-phosphate synthase substitution K284N on Muscat flavour formation. J. Exp. Bot. 62, 5497–5508. doi: 10.1093/jxb/err231

Ferrero, M., Pagliarani, C., Novák, O., Ferrandino, A., Cardinale, F., Visentin, I., et al. (2018). Exogenous strigolactone interacts with abscisic acid-mediated accumulation of anthocyanins in grapevine berries. J. Exp. Bot. 69, 2391–2401. doi: 10.1093/jxb/ery033

Martin, D. M., Chiang, A., Lund, S. T., and Bohlmann, J. (2012). Biosynthesis of wine aroma: transcript profiles of hydroxymethylbutenyl diphosphate reductase, geranyl diphosphate synthase, and linalool/nerolidol synthase parallel monoterpenol glycoside accumulation in Gewürztraminer grapes. Planta 236, 919–929. doi: 10.1007/s00425-012-1704-0
